# Financial inclusion for sustainable agriculture: Pathways among smallholder women farmers in rural Zambia

**DOI:** 10.1371/journal.pone.0326980

**Published:** 2025-07-02

**Authors:** Gershom Endelani Mwalupaso, Xianhui Geng, Salman Ibn Yasin

**Affiliations:** College of Economics and Management, Nanjing Agricultural University, Nanjing, PR China; Yangtze University, MALAWI

## Abstract

Rural women constitute a substantial portion of the global agricultural workforce yet remain marginalized in access to credit, agricultural inputs, and information. This persistent exclusion not only constrains their productivity but also undermines efforts to build resilient and sustainable food systems. As climate variability increasingly threatens smallholder farming, climate-smart agriculture (CSA) has gained prominence as a viable approach to enhance agricultural sustainability, productivity, and adaptation. However, adoption of CSA among smallholder women farmers remains patchy and elusive. In response, development actors have promoted village savings and loan associations (VSLAs) to enhance women’s access to financial services. Yet, whether—and through which mechanisms—women participation (WP) in VSLAs influences CSA adoption remains insufficiently understood. To address this knowledge gap, the study investigates the pathways through which WP in VSLAs influences CSA adoption, considering the mediating role of agricultural informatization (AgI). A cross-sectional, mixed-methods design was employed, using a multistage random sampling technique to survey 436 smallholder women farmers in rural Zambia. Quantitative data were collected through a pre-tested structured questionnaire, supplemented by qualitative data from key informant interviews. Mediation analysis, using the Sobel test and bias-corrected bootstrapping, was applied to estimate pathways, while propensity score matching (PSM) was used to check the robustness of results. Findings reveal that WP in VSLAs significantly boosts CSA adoption, with a total effect size of 47%. Notably, 66% of this effect is mediated through AgI, underscoring the critical role of digital tools in supporting agricultural processes and translating financial inclusion into sustainable farming outcomes. By advancing policy discourse on the intersection of financial inclusion, gender equality, and sustainable agriculture, this research provides valuable insights for informing inclusive agricultural policies and development programs aimed at shaping future interventions in global agricultural development.

## Introduction

The global landscape of women’s participation in agriculture has been steadily increasing, with women now playing diverse roles as farmers, unpaid family workers, and laborers in agricultural enterprises [1,2]. As developing economies transition from farming to manufacturing, men are increasingly moving into industrial jobs, leaving agricultural work predominantly to women [3]. This shift is particularly evident in regions such as South Asia, Southeast Asia, and Sub-Saharan Africa (SSA), where women constitute between 60% and 98% of the economically active agricultural workforce [4]. Despite this rising participation, women—especially those in marginalized communities—continue to grapple with intrinsically linked challenges that undermine their ability to achieve long-term agricultural sustainability, including restricted information and knowledge transfer, and heightened vulnerability to climate risks [4–7]. Notably, a major structural barrier that reinforces these challenges is the widespread disparity in agricultural financing [8,9]. According to the 2014 and 2021 World Bank Global Findex reports [database and highlights available at https://www.worldbank.org/en/publication/globalfindex.], nearly half of all farmers globally lack access to essential financial services, and smallholder farmers—particularly women in rural areas—are disproportionately excluded from formal credit systems [10]. This entrenched financing gap underscores a persistent mismatch between the demand for and supply of agricultural credit, ultimately constraining progress toward Sustainable Development Goal (SDG) 13, which calls for urgent action to combat climate change.

Recognizing these challenges, non-governmental organizations (NGOs) have increasingly promoted the institutionalization of informal community-based financial mechanisms—specifically Village Savings and Loan Associations (VSLAs) or village banks [11,12]. These grassroots microfinance groups are designed to provide savings and credit services to rural populations often excluded from formal financial systems. As such, VSLAs have garnered growing attention for their potential to alleviate credit constraints and incentivize the adoption of climate-smart agriculture (CSA), especially in contexts where uptake remains low despite substantial policy advocacy [13–15]. This is particularly critical as climate change intensifies and cultivable land becomes increasingly scarce. Notably, CSA offers both ecological and economic benefits, such as reducing dependency on synthetic inputs, improving natural resource efficiency, lowering input costs, enhancing yields, and increasing household incomes [16–20].

A compelling pathway through which VSLAs may facilitate CSA adoption is agricultural informatization (AgI)—the use of digital tools and services to support agricultural processes—which holds promise for addressing information gaps that have long constrained smallholder productivity [21]. AgI is especially relevant given the persistent limitations of traditional extension systems, often characterized by untimeliness or irrelevance of the information provided, imperfect market data, and the high costs of face-to-face delivery [22]. Since VSLAs offer secure channels for savings, emergency support, and loans tailored to borrowers’ capacity to repay, women farmers are increasingly positioned to afford information and communication technology (ICT)-based innovations—such as mobile-enabled advisories, community radio programs, and televised farming content—enabling access to timely agricultural information, improved decision-making, and enhanced adoption of sustainable practices [23,24]. This trend is reinforced by recent investments in ICT infrastructure, which have significantly improved telecommunications connectivity across Africa. Simultaneously, the declining cost of digital tools—such as mobile phones and radios—has expanded digital access, and AgI is now widely touted as a platform with transformative potential to reach large numbers of women farmers across rural settings [25–27].

Despite the growing recognition of VSLAs as a tool for rural financial inclusion, especially among women, and their effects on agricultural outcomes—such as farm productivity, household income, and economic efficiency [9,28–31]—empirical evidence on their total, direct and indirect effect on CSA adoption remains limited, fragmented, and inconclusive. This study addresses this gap by analyzing data from 436 randomly selected farmers in Zambia to examine the impact of WP in VSLAs on CSA adoption. A novel focus is to examine whether AgI serves as a mediating factor in this relationship. This inquiry is timely and policy-relevant given the central role women play in agriculture and rural development [32–34]. Empowering women through inclusive financial and digital services is critical to addressing structural inequalities and enhancing sustainable agricultural practices [35,36]. From a methodological standpoint, the study improves on past studies by addressing potential bias from observable confounders before conducting mediation analysis—a limitation common in previous research [37,38]. This strengthens the validity of the findings and enhances their utility for both policymakers and practitioners. Overall, this study provides actionable insights for designing gender-sensitive financial programs that support inclusive growth and climate adaptation in agriculture. especially in regions where gender inequality and limited access to finance hinder agricultural productivity and climate adaptation.

The remainder of the article is structured as follows: The next section provides a comprehensive background of the study, followed by the materials and methods section. This is succeeded by the empirical results and discussion section, and, finally the conclusion, policy and practical implications section.

## Background

### Country context

In Zambia, women constitute the backbone of rural agriculture, making up a substantial portion of the labor force and playing a key role in household food production and rural livelihoods [39,40]. Despite their critical contribution, women face persistent barriers in accessing productive resources, including land, credit, and information services. This challenge is compounded by the broader structural limitations of Zambia’s agriculture sector, which, although contributing about 40% to national GDP and employing over 80% of the population, remains underfunded and heavily reliant on smallholder, rain-fed farming systems [41]. Notably, financial exclusion continues to be a major constraint. Less than 10% of formal bank lending targets agriculture, with rural and women farmers particularly underserved due to weak infrastructure and limited outreach [42,43]. As a result, many farmers increasingly rely on informal mechanisms such as VSLAs, although robust empirical evaluations of their effects on credit access and welfare outcomes are still lacking [42,44].

Though the country is susceptibility to adverse weather conditions, the adoption of sustainable agricultural practices (SAPs) among smallholder women farmers is relatively low. A critical barrier is the lack of access to relevant and timely information. Traditional extension approaches, such as the “Training and Visit” system, are often prohibitively expensive and unsustainable given budgetary constraints [45]. Moreover, agricultural extension services suffer from weak political support, resulting in limited coverage and inadequate reach, particularly among small-scale farmers in low- and middle-income settings [46]. Geographic isolation further compounds the challenge, as vast rural areas remain underserved. Even when extension services are available, a mismatch often exists between the information disseminated and the actual needs and contexts of farmers, reducing the relevance and effectiveness of advisory services. These systemic challenges collectively constrain the widespread adoption of sustainable practices in smallholder systems.

Concurrently, ICTs have witnessed substantial penetration across various regions, emerging as crucial tools for empowering smallholder farmers by enhancing decision-making capabilities to improve yields, incomes, and resilience. This shift is particularly important as traditional farming approaches, relying on farmers’ experience, indigenous knowledge, and guesswork, are proving insufficient to address the growing complexities of modern agricultural challenges. While the adoption of digital tools has provided farmers with more accurate and timely access to information, enhancing their capacity to respond to changing conditions, digital agricultural initiatives in the Zambia are largely driven by non-state actors. These initiatives often suffer from limited coordination and insufficient government support, which reduces their scalability and impact [47]. Moreover, the absence of a comprehensive national policy on digital agriculture exacerbates the challenge of fully leveraging digital tools for agricultural transformation.

### The making agriculture a business (MAB) project

The MAB project was implemented from 2017 to 2022 in Zambia’s Central Province through a partnership led by Christian Aid and Norwegian Church Aid, with funding from the Scottish Government. Operating in Chisamba, Kapiri Mposhi, Mumbwa, and Kabwe districts, the project aimed to shift smallholder agriculture from subsistence to market-oriented farming by improving market engagement, financial access, and climate resilience among rural households.

At its core, the project promoted the formation of VSLAs. Governed by internal constitutions that defined leadership roles, savings contributions, loan terms, and repayment guidelines, VSLAs operated on the basis of voluntary membership, regular savings, and transparent lending practices. Each group comprised up to 30 members who met regularly to contribute savings and manage loans. The model enabled members—especially women—to access small, collateral-free loans for household and agricultural investments. Some groups also received seed capital from implementing organizations to strengthen their lending capacity and long-term sustainability.

MAB placed strong emphasis on gender equality and social inclusion, prioritizing participation by women, youth, and persons with disabilities. Women constituted the majority of VSLA members and played active roles in governance, agricultural learning, and leadership development. Demonstration and incubation sites served as platforms for technical training and peer learning, with over 80% of participants in the demonstration gardens being women.

To strengthen productivity and adaptive capacity, the project advanced sustainable intensification through the promotion of CSA practices grounded in technical efficiency, environmental sustainability, and social acceptability. These included soil and water conservation, agroforestry, crop rotation, integrated pest management, and improved seed use. The project also introduced solar-powered irrigation systems and low-water-use drip irrigation kits, negotiated with suppliers to enhance affordability and access. These innovations extended the production season, reduced climate-related risks, and improved both environmental outcomes and farm profitability.

Although not explicitly framed as a digital agriculture initiative, MAB contributed to agricultural informatization by integrating mobile-based financial services, solar energy infrastructure, and market-access technologies. Mobile money platforms were promoted to facilitate savings, credit access, and reinvestment—particularly for women and youth—while familiarizing farmers with mobile tools and digital transaction tracking. Although the project did not implement dedicated ICT advisory systems, it laid important groundwork for digital integration by supporting cooperative models with embedded business and information-sharing functions, and enhancing access to solar-powered cold storage and real-time market linkages.

Overall, the MAB project offered a comprehensive and inclusive model for agricultural development—blending financial empowerment, digital innovation, and climate resilience within a community-led framework. Its emphasis on women’s agency, collective action, and locally driven knowledge exchange created an enabling environment for transforming rural livelihoods in sustainable and equitable ways.

### Conceptual framework

**Fig 1:**
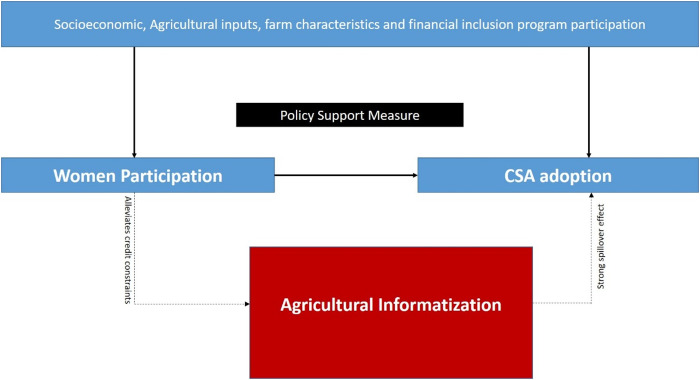
Conceptual framework.

The study is anchored in two key theoretical perspectives: the diffusion of innovations [48] and the induced innovation theory [49]. The former emphasizes that the rate of adoption of an innovation is influenced by specific characteristics, such as trialability and relative advantage. These attributes determine how quickly and widely an innovation spreads among potential adopters. On the other hand, the induced innovation theory highlights that household-level constraints and resource endowments—such as wealth, labor availability, and credit access—play a pivotal role in shaping innovation adoption decisions. Drawing on these theoretical foundations, as illustrated in Fig 1, we posit that WP in VSLA exerts both direct and indirect effects on the adoption of CSA.

#### Direct effect.

WP in VSLA directly enhances CSA adoption by improving access to credit, enabling households with WP to invest in CSA technologies and practices.

#### Indirect effect.

WP in VSLA is hypothesized to indirectly promotes CSA adoption by alleviating credit constraints and enabling the uptake of ICT to support agricultural processes. This, in turn, supports knowledge sharing, better decision-making, and increased efficiency in CSA implementation. The indirect effect is expected to be stronger than the direct effect, as ICTs play a transformative role in scaling CSA practices through timely, context-specific, and accessible information. Moreover, AgI may generate positive spillover effects, where non-participating farmers in vibrant VSLA areas benefit from improved information flows, peer learning, and exposure to agricultural demonstrations—contributing to wider adoption of CSA practices.

This framework underscores the interconnected roles of financial inclusion, digital transformation, and knowledge dissemination in fostering sustainable agricultural [50,51].

## Materials and methods

### Data

The study targeted smallholder women farmers in the four districts where the MAB project was implemented—namely Chisamba, Kapiri Mposhi, Mumbwa, and Kabwe—in Zambia’s Central Province. A descriptive research design with a quantitative approach was employed, and data collection was conducted between June 21 and July 30, 2022.

**Fig 2:**
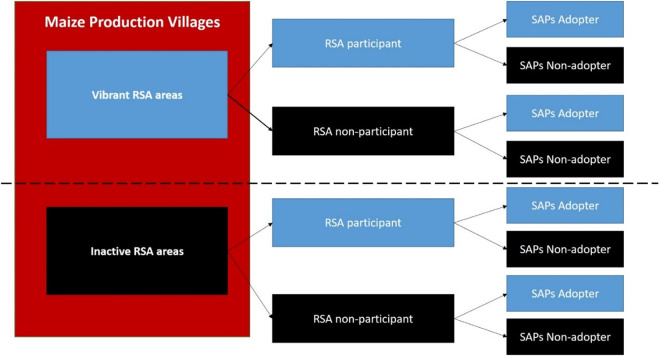
Selection and distribution of sample.

A multi-stage sampling technique was used to select the sample of farm households. In the first stage, two agricultural villages in each district were purposively selected in consultation with local leaders and community members. These villages were matched based on similar socio-economic and infrastructural characteristics, including predominant crop and livestock activities, access to markets and extension services, land tenure arrangements, infrastructure (e.g., roads, water, electricity), climate conditions, and social networks. Within each selected village, two groups were defined based on the intensity of MAB-related activities as shown in Fig 2: villages with active MAB interventions were categorized as “vibrant VSLA areas”, while those with limited or no MAB presence were designated as “inactive VSLA areas”. This stratification helped minimize potential program placement bias, consistent with the approach suggested by Tambo and Wünscher [52]. Within each selected village, two groups were defined based on the intensity of MAB-related activities: villages with active MAB interventions were categorized as “vibrant VSLA areas”, while those with limited or no MAB presence were designated as “inactive VSLA areas”. This stratification helped minimize potential program placement bias, consistent with the approach suggested by Tambo and Wünscher (2018).

From each village, a list of women farmers registered with the local village leadership was used to randomly selected respondents. In cases where multiple women from the same household were selected, a screening process was used to determine whether they managed separate or shared farm plots. If each respondent managed a distinct plot, both were retained in the sample; however, if the plot was shared, only one respondent was included, selected by mutual agreement within the household. To ensure the sample’s representativeness, sample size calculations were conducted following the criteria by Cochran [53] for known population sizes (Where *n* = Sample size, *N* = Population size (3600), *Z* = Z-value (1.96), *p* = Estimated proportion of an attribute present in the population (50%) and *E* = Margin of error (5%)). This yielded a required sample size of 450 farm households. However, only 436 completed surveys were retained for analysis due to missing or incomplete responses. The distribution aimed for 300 households from vibrant areas and 150 from inactive areas, maintaining a proximity of approximately 35–40 kilometers between the two groups. Ultimately, the sample consists of 289 households with WP (60% were from vibrant VSLA areas and 10% from inactive VSLA areas) and 149 non-participants (5% were from vibrant VSLA areas and 25% from inactive VSLA areas).

A structured questionnaire was developed specifically for this study in English and later translated into local languages (primarily Bemba and Nyanja) to ensure clarity and cultural relevance. The translations and administration were conducted by experienced and well-trained enumerators fluent in both English and the respective local languages. The questionnaire was pretested among smallholder women farmers in Kabwe District, one of the study sites selected due to its agro-ecological and socio-economic similarity to the other districts, making it a suitable environment for identifying ambiguities, testing clarity, and refining question structure. Feedback from the pretest informed necessary adjustments to improve comprehension and flow. The data collection tool consisted primarily of closed-ended questions covering key domains such as demographics, farm characteristics, agricultural production, access to finance, VSLA participation, and adoption of agricultural practices (S1 File). Responses included binary choices, Likert-type scales, and categorical formats, depending on the variable type. Where applicable, responses were later recoded or transformed into indices or dummy variables for analysis.

To minimize measurement error, the finalized questionnaire was administered between June 21 and July 24, 2022 by trained enumerators using a digital data entry system. Additionally, key informant interviews (KIIs) were conducted with project staff, agronomists, training center facilitators, and lead farmers to triangulate findings and enrich the interpretation of results. To mitigate potential recall bias, data were collected with reference to the immediate past farming season (2021/2022), consistent with established best practices by Tarrant, Manfredo [54] and Connelly, Brown [55]. All survey data were securely stored and later accessed for analysis on October 31, 2022.

Table 1 presents the summary statistics of the sample, revealing significant differences in key characteristics between households with and without WP in VSLAs. Notably, women who do not participate in VSLAs tend to have less farming experience and smaller landholdings compared to their participating counterparts. These disparities underscore importance of accounting for observable characteristics or the potential for selection bias if one relies on naïve estimators, which may wrongly attribute differences in CSA adoption to WP without accounting for underlying heterogeneity. Observable characteristics refer to household and farm-level variables that may simultaneously influence both the likelihood of joining a VSLA and the adoption of CSA practices. These characteristics were carefully selected based on both theoretical and empirical relevance to VSLA participation and CSA adoption. They include: landholding size, gender, age, marital status, market access, type of farm housing, education level, farming experience, main crop cultivated, access to agricultural extension, presence of a demonstration site, distance to financial institutions (FinsDist), and family size.

**Table 1:** Summary statistics of households based on WP.

Variable	Description	Mean		Difference	SE
		No WP (N = 149)	WP (N = 287)		
CSA	Dummy: 1 = Household adopts CSA	0.29	0.92	−0.63^***^	0.04
AgI	Dummy: 1 = Household adopts AgI	0.10	1.00	−0.90^***^	0.02
Land	The area cultivated to maize (hectares)	1.50	1.95	−0.45^***^	0.11
Gender	Dummy: 1 = Household head is female	0.12	0.08	0.04	0.03
Age	Age of household head (years)	44.10	45.29	−1.19	0.88
Marital	Dummy:1 = Household head is married	0.91	0.95	−0.04	0.03
Market	Distance from homestead to market (kilometers)	1.98	1.92	0.06	0.11
FarmHouse	Distance from homestead to farm plot	0.52	0.53	0.00	0.05
Education	Dummy: 1 = Household head can read and write	0.55	0.51	0.04	0.05
Experience	Farming experience of household head (years)	18.14	23.56	−5.43^***^	1.05
Main crop	Dummy: 1 = Maize is the main crop	0.86	0.96	−0.11^***^	0.03
Extension	Dummy: 1 = Household has access to extension service	0.60	0.67	−0.07	0.05
Demosite	Dummy: 1 = Adult member of household participates at demosites	0.45	0.46	−0.01	0.05
FinsDist	Distance from homestead to nearest formal financial institution (kilometers)	4.33	4.09	0.24	0.16
Family size	Number of people in a household	5.98	5.77	0.21	0.14

**Notes:** Figures in parenthesis are standard errors of the coefficients.

*** p < 0.01,

** p < 0.05,

* p < 0.1

Ethical approval for this study was obtained from the Research and Ethics Committee at Prince G Consultancy and Academy—an independent research and training institution that operates in accordance with national ethical standards for social science research—, certified under number PGCA_SFI/2022513 on June 12, 2022. The approval process involved submitting a detailed two-page concept note, outlining the study’s background, objectives, and anticipated contributions. The ethics committee granted permission for researchers to obtain participant consent verbally, with an emphasis on recording the consent in the presence of the local village leadership representative who was part of the research team. Additionally, researchers diligently adhered to all research guidelines when interacting with human participants, prioritizing the safety and well-being of both researchers and participants.

During data collection, experienced and trained enumerators provided clear explanations of the study’s subject matter to participants, who were assured of confidentiality and informed that they were not required to disclose their names on any part of the questionnaire or during interviews. Furthermore, participants were informed of their right to withdraw from the study at any point if they felt uncomfortable with the questions or the data collection process. Moreover, authors had no access to information that could identify individual participants during or after data collection. This approach ensured the collection of reliable and ethically sound data while minimizing potential risks to participants.

### Operationalization of key variables

In this study, WP in VSLA, is defined as a dummy variable measured at the household level. It takes a value of 1 if at least one woman adult in the household participated in VSLA and 0 otherwise.

Likewise, CSA adoption is captured as a dummy where 1 represents a household that has adoption at least any of the CSA practices promoted in MAB (drip irrigation, improved seed, conservation farming and crop rotation with legumes) and 0 otherwise.

AgI is captured as a dummy variable, with 1 representing a household where an adult member of a household used ICTs (mobile phone, radio and TVs) to support agricultural processes and 0 otherwise.

### Empirical strategy

The study employs mediation analysis to estimate the total, direct, and indirect effects of WP in VSLA on CSA adoption. Additionally, propensity score matching (PSM) is applied to derive a matched sample as well as calculate the total effect using three matching algorithms- nearest neighbor (NNM 1), kernel (KM) and radius (RM) for robustness checking.

### Mediation analysis

Guided by Taylor, MacKinnon [56] and Hayes [57], with the hypothesis that outcome variables are the linear function of the WP in VSLA and other control variables, the direct and indirect effects of WP in VSLA on CSA adoption can be defined as:


$${CSA}_{i}={WP}_{i\;}\delta_{i}+\;X\beta_{i}+\;e_{1}$$ (1)

$${AgI}_{i}={WP}_{i\;}{ℵ}_{i}+\;X\beta_{i}+\;e_{2}$$ (2)

$${CSA}_{i}={WP}_{i\;}\delta_{i}^{\prime }+{AgI}_{i\;}\partial_{i}+\;X\beta_{i}+\;e_{3}$$ (3)

where ${CSA}_{i}$ is representing CSA adoption and ${WP}_{i\;}$is also a dummy where 1 represents an adult woman in a farm household participating in VSLA. The mediation variable, ${AgI}_{i}$, is defined as dependent variables in equations (2), but independent variable in equation (3) as important factors affecting CSA adoption. $\delta_{i}$ is total effect (TE) of WP in VSLA on CSA adoption, and $\delta_{i}^{\prime }$ is the direct effect (DE) of WP in VSLA after controlling for the mediator and other control variables. X is a vector of observable exogenous factors. $\beta_{i}$, ${ℵ}_{i}$ and $\partial_{i}$ are the estimated coefficients of the relevant independent variables. Finally, $e_{1}$, $e_{2}$, and $e_{3}$ are error terms.

To accurately determine the causal influence of WP in VSLA on CSA adoption, farmers would ideally be randomly assigned into two groups: those with WP in VSLA and those without. However, since WP in VSLA is not randomly assigned and households self-select into participation, directly estimating the model may result in biased estimates due to selection bias. To ensure robust and reliable estimates, several modeling decisions must be made to account for this potential bias. First, a subsample was derived by employing a “1-to-1 nearest neighbor matching without replacement” approach, offering two key advantages: (i) it ensures the condition of common support by matching every adopter with a non-adopter [58], and (ii) it provides the most intuitive interpretation and is easy to implement compared to alternative methods [59]. Accordingly, based on the original sample of 287 women farmers who participated in VSLAs and 149 who did not, matching was limited by the number of available control units, resulting in 149 matched pairs. This yielded a balanced subsample of 298 observations—149 treated and 149 matched controls—referred to as the matched sample. The original set of 436 observations prior to matching, comprising all participants and non-participants, is referred to as the pooled sample. These categories are used consistently in subsequent analyses, tables, and figures.

Ideally, the first assumption, known as the conditional independence assumption (CIA), posits that given a set of observable covariates (X), the potential outcome (Y = CSA adoption) is independent of the treatment (T = WP in VSLA) allocated. Mathematically, this is expressed as:


$${(\text{Y}}_{1}),\;({\text{Y}}_{0})⊥\;\text{T}|\text{X}$$ (4)

The second assumption is that perfect predictability is prevented by overlap or common support; this is expressed as:


0<P (T=1|X)<1 (5)

We estimate the likelihood of WP participation using a probit model, from which propensity scores are derived. These scores represent the probability (ranging from 0 to 1) of a woman farmer participating in VSLAs, conditional on observable characteristics outlined in the Data subsection of the Materials and methods section. The propensity scores are then used to match women in the treatment group (those with WP in VSLAs) with those in the control group (non-participants), ensuring comparisons are made within a region of common support.

The concept of common support is central to matching as it strengthens the internal validity of causal inference by restricting comparisons to the region where treated and control observations share similar characteristics [59,60]. To ensure the common support condition was satisfied and covariate balance was achieved, a visual assessment of the distribution of estimated propensity scores was performed using histograms. This assessment helps to determine whether the treated and control groups share a region of overlap in their propensity scores. Observations with propensity scores lying outside this region—meaning they have no suitable match—were identified as off-support and excluded from the analysis. Off-support observations typically reflect households whose observable characteristics are so distinct that meaningful comparison is not possible, thus posing a risk of extrapolation bias. This exclusion improves the credibility of the treatment effect by restricting inference to comparable units—control and treated groups.

Following verification of the common support condition, the next step involved assessing covariate balance and the extent of bias reduction achieved through matching. Here, bias refers to the standardized mean differences in observed covariates between treated and control groups. Two visual diagnostic tools were used to evaluate this. First, a kernel density plot of the estimated propensity scores was generated to compare the overall distribution of treatment probabilities between groups, before and after matching. After matching, closer alignment of the density curves indicates that treated and control units share more similar covariate structures, signifying a reduction in selection bias and improved comparability. Second, a box plot summarizing the distribution of standardized mean biases across all covariates was used to further assess balance. The box plot reveals substantial covariate imbalance before matching, reflected in wider spreads and distinct medians between groups. After matching, if the boxes and medians align closely, it signals successful bias reduction and enhanced covariate balance. Together, these plots validate the effectiveness of the matching procedure in minimizing differences in observable characteristics between the two groups, ensuring that comparisons are more meaningful and accurate.

Second, to uncover the pathway through which WP influences the adoption of CSA, we apply mediation analysis. Common statistical techniques for testing mediation effects include causal steps tests (CST), difference in coefficients tests (DCT), and product of coefficients tests (PCT) [61,62]. CST, introduced by Baron and Kenny [63], is widely used for its simplicity but is limited by its requirement for a significant direct effect and low statistical power [61,64]. DCT, which compares regression coefficients before and after including the mediator, may suffer from inflated Type I error rates in complex models [61,65]. In contrast, PCT is more robust and statistically powerful but relies on the Sobel method [66], which assumes normality—a condition often violated in practice. To address these limitations, bootstrapping techniques, which do not rely on normality and are effective with various sample sizes, are preferred [56,61]. Specifically, the bias-corrected bootstrap method provides improved accuracy for confidence intervals. Therefore, in this study, we utilize the bias-corrected bootstrapping approach to examine the direct, indirect, and total effects of WP on CSA adoption among maize farmers in rural farm households, with AgI as the mediating variable. This approach efficiently manages mutual causality by enabling the simultaneous estimation of interrelated equations, significantly reducing bias and accounting for interdependencies by treating these relationships as a system.

### Propensity score matching

To ensure the robustness of the estimated impact of WP in VSLA, PSM was applied to estimate the treatment effects using multiple indicators. Specifically, we compute the Average Treatment Effect on the Treated (ATT), the Average Treatment Effect (ATE), and the Average Treatment Effect on the Untreated (ATU), with standard errors bootstrapped using 1,000 replications. In addition, a sensitivity analysis is conducted to assess the reliability of the estimated effects under potential violations of the conditional independence assumption.

PSM is widely utilized in impact assessments. Given its extensive coverage in existing literature and its secondary role in this analysis, only a concise description of its formal proof is provided here. For foundational insights into PSM, readers are referred to the seminal work by Rosenbaum and Rubin [67] and the numerous applications that followed.

### Empirical results and discussion

This section presents the empirical results and a succinct discussion of the findings.

### Impact of WP in VSLA on CSA

**Fig 3:**
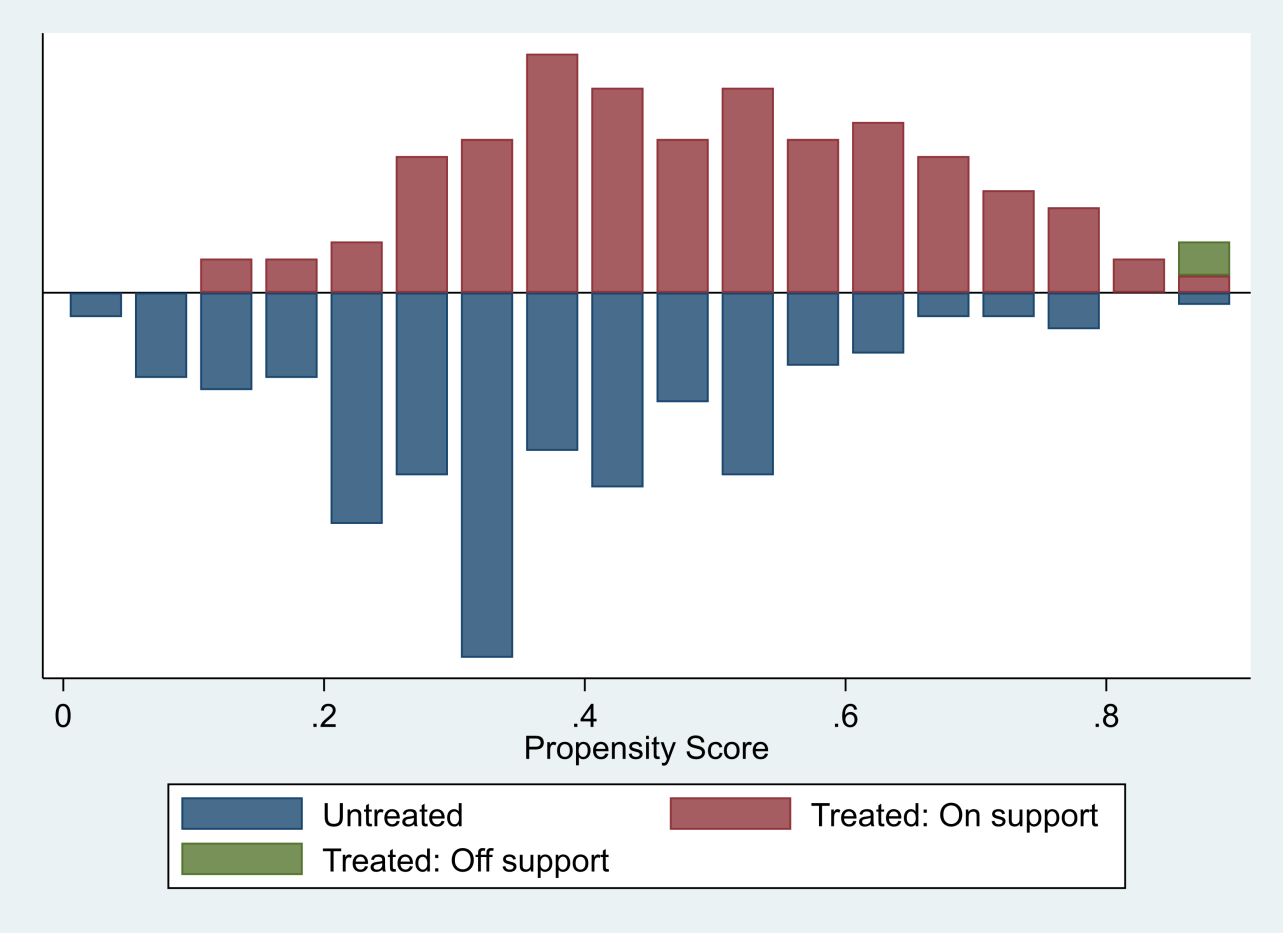
Balancing of covariates achieved imposing a region of common support.

**Fig 4:**
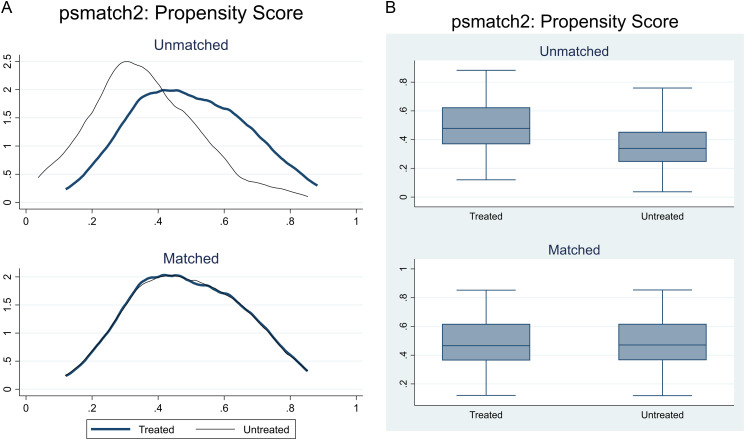
(a) Kernel density distribution of bias reduction. (b). Box plot distribution of the bias reduction.

Table 2 presents the mediation analysis results for both the pooled and matched samples. Before delving into the findings, it is important to highlight the quality of matching, demonstrated in Fig 3 and 4. The former shows that a region of common support was imposed, with only two observations falling outside the common support region. Additionally, in Fig 4a and 4b, there was a significant reduction in bias across observable characteristics, enhancing the comparability of the two groups.

**Table 2:** Estimation results of WP, AgI and CSA adoption.

Explanatory variables	Pooled						Matched					
	CSA (1)		AgI (2)		CSA (3)		CSA (4)		AgI (5)		CSA (6)	
	Coef	SE	Coef	SE	Coef	SE	Coef	SE	Coef	SE	Coef	SE
WP	0.612^***^	0.032	0.902^***^	0.022	0.218^***^	0.069	0.473^***^	0.042	0.865^***^	0.032	0.161^**^	0.076
AgI					0.437^***^	0.069					0.361^***^	0.075
Land	−0.040^***^	0.015	−0.010	0.010	−0.036^**^	0.014	−0.040^**^	0.017	−0.013	0.013	−0.036^**^	0.017
Gender	−0.049	0.052	−0.037	0.035	−0.033	0.050	−0.080	0.080	−0.068^*^	0.041	−0.062	0.077
Age	0.013^***^	0.002	−0.021^***^	0.001	0.012^***^	0.002	0.013^***^	0.003	0.001	0.002	0.013^***^	0.003
Marital	−0.004	0.062	−0.041	0.042	0.013	0.059	−0.010	0.103	−0.113	0.079	0.031	0.100
Market	0.036^**^	0.017	0.021^*^	0.011	0.031^*^	0.016	0.038^*^	0.022	0.028^*^	0.017	0.037^*^	0.021
FarmHouse	0.049	0.031	0.015	0.021	0.043	0.029	0.069^*^	0.036	0.017	0.027	0.063^*^	0.034
Education	−0.012	0.034	−0.017	0.023	−0.005	0.032	−0.040	0.043	−0.028	0.033	−0.029	0.042
Experience	0.002	0.002	0.001	0.001	0.001	0.002	0.001	0.002	0.002	0.002	0.001	0.002
Main crop	0.059	0.052	0.039	0.035	0.042	0.050	0.097^*^	0.057	0.059^*^	0.031	0.076^*^	0.045
Extension	−0.065^***^	0.032	−0.061^***^	0.022	−0.038	0.031	−0.050	0.040	−0.078^**^	0.030	−0.022	0.039
Demosite	0.096^***^	0.031	0.102^***^	0.021	0.051^*^	0.031	0.068^*^	0.041	0.129^***^	0.031	0.022	0.040
FinsDist	−0.071^***^	0.011	−0.010	0.007	−0.067^***^	0.010	−0.097^***^	0.015	−0.008	0.011	−0.094^***^	0.014
Family size	0.034^***^	0.012	0.019^**^	0.008	0.026^**^	0.012	0.042^***^	0.015	0.022^*^	0.011	0.035^**^	0.014
Constant	−0.252^***^	0.132	−0.044	0.089	−0.233^*^	0.126	−0.185	0.197	−0.018	0.150	−0.179	0.189
R-square	0.612		0.824		0.646		0.535		0.757		0.570	
N	436						289					

Notes:

*** p < 0.01,

** p < 0.05,

* p < 0.1. Coef refers to the estimated coefficient, while SE represents the standard error of the estimates.

Having confirmed that a good match was achieved, we now turn our attention to the results presented in Table 2, which are consistent across both the pooled and matched samples. However, greater emphasis is placed on the matched sample results, as they effectively address bias stemming from observable characteristics. In contrast, the pooled sample results are prone to overestimation or underestimation due to these biases. For instance, the total effect of women’s participation (WP) in VSLA on CSA adoption is approximately 61% in the pooled sample and 47% in the matched sample (column 1 and 4, Table 2). In both models, the effect is positive and statistically significant at the 1% level. However, after accounting for the mediating variable (Column 3 and 6, Table 2), the effect decreases to 22% in the pooled sample and 16% in the matched sample. This reduction is plausible, as women’s participation in agricultural activities has been instrumental in driving technology adoption, even in contexts where women are often marginalized in access to resources and decision-making power [35,68]. Moreover, women are known to have strong social networks, which can amplify the dissemination of agricultural knowledge and practices, further driving CSA adoption [69].

Likewise, in Columns 3 and 6 of Table 2, the results indicate that AgI has a strong positive impact on CSA adoption, with effects estimated at 44% in the pooled sample and 36% in the matched sample. Notably, this impact is nearly twice as large as the direct effect of WP on CSA adoption, underscoring the transformative potential of digital tools in agriculture.

Finally, the results in Columns 2 and 5 of Table 2 demonstrate that WP significantly enhances AgI adoption, with an estimated effect of 90% in the pooled sample and 85% in the matched sample. This strong association underscores that WP in VSLAs does more than improve financial security—it facilitates greater engagement with agricultural information tools such as radio, TV, and mobile phones.

While the use of these devices—particularly mobile phones and radios—often occurs at the individual level on farms, participation in VSLAs plays a catalytic role. Through group meetings, women gain exposure to new ideas, exchange tips on where and how to access information, and are encouraged by their peers to apply these tools in their farming practices. These meetings sometimes include informal discussions around CSA-related radio broadcasts, agricultural TV segments, or mobile-based advisories, serving as an entry point for women to seek out and trust digital agricultural services as observed by a key informant. Moreover, radio remains especially valuable in rural Zambia, where low literacy levels make it a practical and accessible source of agricultural knowledge. Women in VSLAs, through pooled savings and strengthened financial autonomy, are more likely to afford basic devices such as radios or feature phones. TV, though less widespread, also plays a role where accessible—especially when women gain more voice in household purchasing decisions through their group participation.

Interestingly, mobile phones, in particular, are becoming a transformative tool for AgI. Through VSLA participation, women are increasingly able to purchase or share mobile devices, which enable them to receive weather alerts, market price updates, and direct communication with extension services via SMS or voice calls. More importantly, phones support peer-to-peer learning within and beyond the VSLA structure—allowing women to consult each other, share best practices, and ask for guidance on CSA techniques. This informal network strengthens both the uptake and practical application of AgI tools on their farms [70].

However, structural limitations still constrain the full potential of AgI. According to a key informant, limited seed capital remains a major barrier. Many women farmers’ savings through VSLAs are still modest, and without reliable market linkages, they struggle to translate information into productive investment. In addition, there is a shortage of adequate, context-specific, and culturally sensitive content on these AgI platforms. Much of the information disseminated via radio, TV, or mobile channels lacks local relevance or fails to resonate with the lived realities of rural women. As noted by the key informant, this disconnect can reduce trust and engagement with digital tools, ultimately limiting their impact on CSA adoption. This constraint highlights the importance of coupling AgI with broader ecosystem support—such as improved market access, tailored content development, and stronger input systems—to ensure that the information received is both actionable and meaningful.

In essence, VSLAs create an enabling environment where women farmers are better positioned to access and apply agricultural information individually, while also fostering a culture of information-sharing and mutual encouragement. As such, VSLAs function not only as financial platforms but also as critical stimulators of AgI, strengthening women’s ability to adopt CSA practices and adapt to climate challenges.

### Mechanism of the impact of WP in VSLA on CSA

The decomposition of the effect of WP into direct, indirect, and total effects is presented in Table 3, with results showing consistency across both the bias-corrected bootstrap method and the Sobel test. The direct effect of 16% is plausible given the multiple benefits that participation in VSLA offers to farmers. Firstly, VSLA enhance access to credit, especially in rural areas where formal banking services are limited. Secondly, the flexibility of borrowing funds based on individual needs and repayment capacity enables farmers to invest in sustainable practices at critical times. Thirdly, savings groups operate on a smaller scale with lower overhead costs, making credit more accessible and affordable to farmers. Finally, participation in savings groups contributes to the development of financial management skills, including budgeting, saving, and debt management. By acquiring these skills and building a positive credit history within the group, farmers are better equipped to adopt CSA practices, make informed investment decisions, and manage resources efficiently [29].Overall, savings groups empower farmers to make productive investments, ultimately fostering resilience and long-term agricultural sustainability within their communities.

**Table 3:** Decomposition of total, direct and indirect effects of WP in VSLA on CSA adoption.

Decomposition	CSA adoption							
	Pooled				Matched			
	Bias Corrected bootstrap		Sobel test		Bias Corrected bootstrap		Sobel test	
	Coef	SE	Coef	SE	Coef	SE	Coef	SE
Indirect Effect	0.394^***^	0.059	0.394^***^	0.063	0.312^***^	0.055	0.312^***^	0.066
Direct Effect	0.218^***^	0.058	0.218^***^	0.069	0.161^***^	0.059	0.161^***^	0.076
Total	0.612^***^	0.033	0.612^***^	0.032	0.473^***^	0.042	0.473^***^	0.042

**Notes:**

*** p < 0.01,

** p < 0.05,

* p < 0.1.

Importantly the proportion of the mediating variable is more than 50% of the total effect in both the pooled and matched sample. This finding aligns with existing literature emphasizing the role of AgI in driving agricultural innovation and enhancing climate change resilience. This finding suggests that AgI—primarily delivered through radio, TV, and mobile phones—plays a critical role in bridging information gaps for smallholder women farmers. By expanding access to timely, practical, and locally relevant agricultural knowledge, these communication tools empower women to make informed decisions, adopt sustainable practices, and better adapt to climate-related challenges. AgI is not merely a technological platform but is widely recognized as a cornerstone for modernizing agriculture, improving competitiveness, and constructing efficient information service systems.

These findings strongly support the conclusion that women’s participation promotes CSA adoption through the mediating role of AgI. Overall, these smallholder financial inclusion programs which are NGO-led such as the MAB, though not always explicitly designed as climate adaptation financing initiatives, still play a critical role in encouraging CSA adoption [71]. Moreover, the presence of the demonstration site, intentionally designed to foster interaction and collaborative learning, served not only as an information hub for sustainable agricultural practices and market linkages but also as a catalyst for AgI among women participants. By promoting the use of mobile phones for information sharing, encouraging access to agricultural content on TV and local community radio, and facilitating the circulation of farming knowledge through social media, the leadership at the demostration site actively stimulated digital engagement. In addition, financial literacy and household-level discussions around managing credit and income was emphasized, reinforcing the importance of joint decision-making and gender equality within farming households. This underscores the idea that improving access to credit alone is insufficient; it must be complemented by targeted efforts to provide timely, relevant information and empower women with the tools and knowledge needed to make informed choices. Such integrated approaches are often underemphasized in credit-focused rural development programs but are vital for enhancing the sustainability and impact of agricultural innovations [72].

However, for AgI to reach its full potential, digital literacy and credit constraints must be addressed, paving the way for wider adoption [73,74]. The effectiveness of AgI in rural Zambia is shaped by complex cultural and structural factors that must be carefully considered. Gender norms often determine control over household technology, frequently privileging men’s access to radios, TVs, and mobile phones, which can limit women’s direct exposure to CSA information. Moreover, literacy constraints and language diversity influence how women receive and interpret messages delivered via these platforms. Even with widespread broadcasting, the usefulness of information depends on its accessibility, clarity, and alignment with local farming conditions.

Trust and social dynamics further mediate the uptake of CSA knowledge. Many rural women may place greater reliance on traditional knowledge, peer networks, and community leaders than on impersonal media sources [75]. If AgI initiatives are seen as externally imposed or disconnected from local realities, their impact may be diminished. Therefore, for AgI to be truly effective, extension programs should prioritize culturally sensitive content, community-driven dissemination methods, and engagement with trusted local figures, thereby ensuring that agricultural information is both credible and actionable [76].

In summary, while the finding highlights the significant promise of AgI in advancing CSA adoption among women farmers, overcoming sociocultural barriers and structural inequalities remains crucial. Aligning AgI strategies with local gender dynamics, educational levels, and farming contexts will maximize their contribution to sustainable rural development and empower women as key agents of agricultural transformation.

### Robustness checking and sensitivity snalysis

The robustness of the findings on the impact of WP in VSLAs on the adoption of CSA was assessed through PSM. The results, as presented in Table 4, demonstrate significant positive effects across different treatment effect measures, highlighting the importance of WP in VSLAs for promoting CSA adoption. Specifically, the ATT reveals a strong positive effect of 68% of WP on CSA adoption among households where women participate in VSLAs. This indicates a substantial impact within the treated group. The ATE extends this impact across the entire sample, suggesting that the benefits of WP in VSLAs for CSA adoption are widespread and not limited to treated individuals alone. Furthermore, the ATU provides a noteworthy perspective, indicating that even households where women do not currently participate in VSLAs would stand to gain considerably from their participation. This finding is crucial as it underscores the potential for scaling VSLAs to benefit a broader population, particularly in enhancing agricultural sustainability in rural areas. The stability of these estimates, supported by the bootstrap standard errors, further reinforces the reliability and robustness of the results. These findings are important because they not only demonstrate the efficacy of VSLAs in fostering CSA adoption among women participants but also highlight their potential to extend positive outcomes to households with non- women participants, should they choose to join. This underscores the broader implications of VSLAs as a scalable intervention capable of enhancing the reach and effectiveness of financial inclusion initiatives aimed at fostering sustainable agricultural practices [11,77].

**Table 4:** Bootstrap estimates for PSM.

Treatment Effect	Coeficient	Bootstrap SE
ATT	0.680^***^	0.056
ATE	0.625^***^	0.039
ATU	0.582^***^	0.050

**Notes:**

*** p < 0.01,

** p < 0.05,

* p < 0.1.

Additionally, we conducted a sensitivity analysis using Rosenbaum bounds for the outcome variable CSA and S1 Table presents the results, with Gamma (Γ) values ranging from 1 to 2, reflecting varying levels of sensitivity to unobserved biases [78]. The sensitivity analysis indicates that the treatment effect on CSA is robust to unobserved biases up to a Gamma value of 2. The upper and lower bound significance levels (sig+ and sig-) remain at 0 across all Gamma values, demonstrating that the results are reliable and significant. Additionally, the Hodges-Lehmann point estimates (t-hat+ and t-hat-) are consistent at 0.5, reaffirming the stability of the estimated treatment effect. The confidence intervals (CI+ and CI-) confirm that the estimated effect size remains stable, with slight variations at higher Γ values. These findings validate the robustness of the treatment effect against potential unobserved confounders, providing strong evidence for the reliability of the estimates.

## Conclusion, policy and practical implications

This study highlights the critical interplay between WP, AgI, and CSA adoption. Specifically, WP exhibits a total effect of 47% on CSA adoption, comprising a 31% indirect effect mediated through AgI and a 16% direct effect. Interestingly, indirect pathway has a greater proportion. Therefore, addressing digital constraints remains central to maximizing the benefits of WP, on CSA, ultimately fostering sustainable agricultural practices and enhancing climate resilience among rural households.

The findings reveal three critical policy implications: enhancing VSLA activities, intensifying hands-on agricultural extension services, and improving AgI. Firstly, AgI plays a pivotal role in redefining and expanding the role of VSLAs within farming communities. Using such platforms to promote the adoption of AgI tools—for mobile banking, access to market information, and knowledge-sharing platforms—can significantly amplify the benefits of financial inclusion and improve the uptake of CSA [79–82]. Moreover, strengthening AgI not only improves the flow of agricultural information but also empowers women farmers to make timely and informed decisions on their farms. Secondly, empowering women through informal financial inclusion mechanisms like VSLAs can facilitate broader gender equality outcomes in agriculture. Enhanced financial autonomy improves their ability to access agricultural inputs, invest in productivity-enhancing technologies, and engage more actively in household decision-making—key enablers for sustainable agriculture. Thirdly, improving market linkages, particularly through contract farming arrangements, can strengthen the incentives for women to participate in VSLAs, adopt AgI tools, and apply CSA practices. Effective market access provides returns on both financial and knowledge investments, thereby encouraging continued participation in VSLAs and sustained agricultural innovation.

On the other hand, the findings offer several practical insights for enhancing WP in VSLAs, AgI, and CSA adoption in Zambia. First, expanding access to VSLAs in underserved communities—particularly through churches and cooperatives—can create more inclusive financial ecosystems, especially for women who face structural barriers to formal credit. Second, AgI efforts can be scaled by integrating ICT training into agricultural extension programs and ensuring that digital tools are accessible in local languages and compatible with basic mobile phones. Partnerships with telecom providers and agritech start-ups can further reduce cost and connectivity barriers. Third, to increase CSA adoption, practical demonstration sites and peer learning platforms should continue to be used to showcase cost-effective and context-relevant practices. Culturally, Zambia’s strong communal norms and high levels of participation in local group-based structures such as women’s clubs and faith-based networks can be leveraged to promote both VSLAs and information-sharing. However, gender norms that limit women’s decision-making power over land use, farming decisions, or technology access may hinder progress. Addressing these cultural constraints through targeted gender-transformative interventions—such as household dialogues or inclusive leadership training—will be critical to achieving sustainable and equitable agricultural development.

Finally, while this study employs a robust empirical strategy, several limitations should be acknowledged. First, the analysis relies on self-reported data, particularly on variables such as farm size, which may be subject to response bias and measurement errors. This limitation may affect the precision of estimated effects, especially where farmers may underreport or overestimate landholding sizes [83,84]. Second, the study does not fully capture the three critical dimensions of AgI—content, connectivity, and capacity. Specifically, there is limited assessment of whether the information disseminated via AgI tools is context-specific, culturally sensitive, and practically useful to women farmers. Similarly, issues of digital connectivity (e.g., mobile network access) and user capacity (digital literacy and comprehension) were not adequately measured, yet these are essential determinants of AgI effectiveness. Third, the study does not estimate the potential spillover effects of AgI—where exposure among some women farmers could influence the knowledge and practices of their peers—and the lagged effects of women’s participation in VSLAs on sustainable agriculture outcomes over time. Understanding these temporal and indirect dynamics would offer deeper insights into the lasting impact and scalability of such interventions. Despite these limitations, the study offers compelling evidence on the effectiveness of WP in VSLAs and AgI adoption in promoting sustainable agriculture. Future research should consider the use of panel data and mixed-method approaches to better capture these overlooked dimensions and explore the long-term impact pathways of financial inclusion and digital agriculture initiatives.

## Supporting information

S1 Table Rosenbaum Bounds sensitivity analysis results. (DOCX)

S1 File Questionnaire. (PDF)
