# Supplementary material for: Financial inclusion for sustainable agriculture: Pathways among smallholder women farmers in rural Zambia
Source: PLoS One. 2025 Jul 2;20(7):e0326980. doi: 10.1371/journal.pone.0326980 (PMC12221078; doi:10.1371/journal.pone.0326980)
Supplement: S1 Table — (DOCX) [file pone.0326980.s001.docx]

**S1 Table. Rosenbaum Bounds sensitivity analysis results**

| Γ | **sig+** | **sig-** | **t-hat+** | **t-hat-** | **CI+** | **CI-** |
| --- | --- | --- | --- | --- | --- | --- |
| 1.0 | 0 | 0 | 0.5 | 0.5 | 0.5 | 0.5 |
| 1.1 | 0 | 0 | 0.5 | 0.5 | 0.5 | 0.5 |
| 1.2 | 0 | 0 | 0.5 | 0.5 | 0.5 | 0.5 |
| 1.3 | 0 | 0 | 0.5 | 0.5 | 0.5 | 0.5 |
| 1.4 | 0 | 0 | 0.5 | 0.5 | 0.5 | 0.5 |
| 1.5 | 0 | 0 | 0.5 | 0.5 | 0.5 | 0.5 |
| 1.6 | 0 | 0 | 0.5 | 0.5 | 0.5 | 0.5 |
| 1.7 | 0 | 0 | 0.5 | 0.5 | 0.5 | 0.5 |
| 1.8 | 0 | 0 | 0.5 | 0.5 | 0.5 | 1.0 |
| 1.9 | 0 | 0 | 0.5 | 0.5 | 0.5 | 1.0 |
| 2.0 | 0 | 0 | 0.5 | 0.5 | 0.5 | 1.0 |

**Notes:** Γ represents the log odds of differential assignment due to unobserved factors. sig+ and sig- refer to the upper and lower bound significance levels, respectively. t-hat+ and t-hat- are the upper and lower bound Hodges-Lehmann point estimates. CI+ and CI- denote the upper and lower bound confidence intervals (95% confidence level), respectively.
