# Supplementary material for: Financial inclusion for sustainable agriculture: Pathways among smallholder women farmers in rural Zambia
Source: PLoS One. 2025 Jul 2;20(7):e0326980. doi: 10.1371/journal.pone.0326980 (PMC12221078; doi:10.1371/journal.pone.0326980)
Supplement: S1 File — (PDF) [file pone.0326980.s002.pdf]

QUESTIONNAIRE FOR FARMERS

I am glad to inform you that you have been randomly selected to participate in a study entitled **Smallholder Financial Inclusion Programs and Agricultural Development.** The primary goal is to understand the nature of savings group and also provide empirical evidence at household level on the benefits of participation. Your responses are strictly confidential and will only be used for academic purposes. Therefore, your participation is highly appreciated.

Researcher

Gershom Endelani Mwalupaso and Team

Instructions: Kindly tick the selected choice or choice(s) in some questions and give the exact or approximate answer in other questions.

The Household head should answer the questions or any knowledgeable member of the household except the information on women participation which should be answered by an adult female in a household.

Date of interview: ..../........./2022

HOUSEHOLD HEAD CHARACTERISTICS (Note: For **Marital Status:** 1 = Married; 2 = Single; 3 = Divorced; 4 = Separated; 5 = Widowed**)**

Names (Optional)	Age	Sex	Marital status<br><br>(**see note below**)	Years of formal education	Years of informal education	Farming experience	Use DT (radio, TV or mobile phone)<br><br>(**1=Yes, 2=No**)	Adopt at least one CSA<br><br>(**1=Yes, 2=No**)	Working hours per day on farm<br><br>(**Hours**)	Income from non-farm activities<br><br>(**Zmw**)	Training experience	Religion<br><br>(**1=Yes, 2=No**)

HOUSEHOLD IDENTIFICATION	2021/2022 MAIN CROP PRODUCTION ON MAIN MAIZE PLOT	SAVING GROUP PARTICIPATION RELATED
Camp name ________________________<br><br>Family size _________________________<br><br>Access to electricity **Yes** [ ] **No** [ ]<br><br>Member of a cooperative **Yes** [ ] **No** [ ]<br><br>Access to extension contact **Yes** [ ] **No** [ ]<br><br>Number of contacts per season **Daily [ ] Weekly [ ] Every two weeks [ ] monthly [ ] Never [ ]**<br><br>Access to financial credit **Yes** [ ] **No** [ ]<br><br>Total land area possessed (Ha) _____________________<br><br>Names of mining company (s) within the area<br><br>____<br><br>**Estimate in Kilometers the**<br><br>Distance from nearest town ≈<br><br>Distance from the market ≈<br><br>Distance from all-weather roads ≈	Maize as main crop<br>____<br><br>Seeds used in kilograms and the price in kwacha<br><br>**Quantity [________________] Price [_________________]**<br><br>Area used in hectares and the price in kwacha<br><br>**Quantity [________________] Price [_________________]**<br><br>Chemical fertilizers used in kilograms and the price in kwacha<br><br>**Quantity [________________] Price [_________________]**<br><br>Chemical Treatment (Herbicides and Insecticides)<br><br>**Quantity [________________] Price [_________________]**<br><br>Labour used during production (first hired and second family)<br><br>**Number days works [_____] Average hours worked/day [_____]**<br><br>**Number days works [_____] Average hours worked/day [_____]**<br><br>Output or production in kilograms and Cost in kwacha<br><br>**Quantity [________________] Cost [_________________]**	Do you participate in savings group? **Yes [ ] No [ ]**<br><br>When did you start participating? ___________________<br><br>Do you have any adult female in this household participating in savings group for the past two years? **Yes [ ] No [ ]**<br><br>Kindly indicate the value of the following held by males and females in the household (ZMW)<br><br>**agricultural equipment (hoes, saws, wheelbarrows, sprayers, etc.)**<br><br>**male ( ) Females ( )**<br><br>**vehicles (bikes, motorbikes, trucks)**<br><br>**male ( ) Females ( )**<br><br>Did you have any prior knowledge on RUSACCO before joining? **Yes [ ] No [ ]**

Distance from the farm to home ≈		

Agricultural Informatization

Do you own any of the following devices? (Multi-select)

Mobile phone, Radio, Television, None

Do you have neighbors who own any of the following devices? (For each unchecked option above)

Radio: [ ] Yes [ ] No, TV: [ ] Yes [ ] No , Mobile phone: [ ] Yes [ ] No

Do you use these devices to access agricultural information? (Multi-select)

Mobile phone (If yes, proceed to Q5), Radio (If yes, proceed to Q6), TV (If yes, proceed to Q7), None

How long have you used these devices for agricultural information?

Mobile phone: [ ] <1 year [ ] 1–3 years [ ] >3 years [ ] Never

Radio: [ ] <1 year [ ] 1–3 years [ ] >3 years [ ] Never

TV: [ ] <1 year [ ] 1–3 years [ ] >3 years [ ] Never

What agricultural processes does it support? (Multi-select)

Weather forecasts, Market prices (inputs/outputs), Extension advice (e.g., pest control, planting dates), Mobile money/credit services, Labor/input availability, Cooperative updates, Other: ________

How frequent do device

Not useful [ ] Somewhat useful [ ] Useful [ ] Very useful

Rate the signal quality for these devices in your area:

Mobile network: [ ] Poor [ ] Fair [ ] Good [ ] Excellent

Radio signal: [ ] Poor [ ] Fair [ ] Good [ ] Excellent

TV signal: [ ] Poor [ ] Fair [ ] Good [ ] Excellent

Challenges in using these devices: (Multi-select per device)

**Challenge**	Mobile	Radio	TV
High costs (airtime/electricity)	[ ]	[ ]	[ ]
Poor signal/reception	[ ]	[ ]	[ ]
Lack of relevant programs	[ ]	[ ]	[ ]
Language barriers	[ ]	[ ]	[ ]
Difficulty operating device	[ ]	[ ]	[ ]
Other: ________	[ ]	[ ]	[ ]

Prior knowledge of operating these devices:

No experience [ ] Self-taught [ ] Received training

Do you receive agricultural information from these sources via phone/radio/TV? (Multi-select)

Government extension (), NGOs/projects, Farmer groups, Other: ________

Rate the importance of these devices for farming:

Mobile phone: [ ] Not important [ ] Somewhat [ ] Important [ ] Very important

Radio: [ ] Not important [ ] Somewhat [ ] Important [ ] Very important

TV: [ ] Not important [ ] Somewhat [ ] Important [ ] Very important

Benefits of using these devices: (Multi-select)

Saves time/travel costs, Improves access to timely info, Increases yields/income, Enhances social connections, Other: ________

How often do you access agricultural information through these channels?

Radio: Daily [ ] Weekly [ ] Monthly [ ] Rarely [ ] Never [ ]

TV: Daily [ ] Weekly [ ] Monthly [ ] Rarely [ ] Never [ ]

Mobile Phone: Daily [ ] Weekly [ ] Monthly [ ] Rarely [ ] Never [ ]

What challenges do you face in using radio for agricultural information? (Tick all that apply)

Poor radio signal in my area [ ] Lack of relevant agricultural programs [ ] Language barriers [ ] No access to a radio set [ ] Programs are broadcast at inconvenient times [ ]

What challenges do you face in using TV for agricultural information? (Tick all that apply)

No access to electricity/power supply [ ] Agricultural programs are not available in my area [ ] High cost of owning a TV [ ] Language barriers [ ] Agricultural programs are aired at inconvenient times [ ]

What challenges do you face in using mobile phones for agricultural information? (Tick all that apply)

Funds for recharging airtime [ ] Network problems [ ] Power for charging the battery [ ] Complexity of mobile phone technology [ ] Absence of an information center [ ] Language barriers [ ]

Which of the following changes have you made in your farming as a result of information received from radio, TV, or mobile phone? (Tick all that apply)

Adopted new planting techniques [ ] Improved pest and disease control methods [ ] Adjusted planting schedule based on weather updates [ ] Changed crops based on market price updates [ ] Increased farm productivity [ ]

How often do you use your mobile phone to access agricultural information during the planting season?

Daily [ ] Weekly [ ] Once per month [ ] Others (Specify) _______________

How has access to agricultural information through radio, TV, or mobile phone benefited you? (Tick all that apply)

Helps to easily send and receive timely agricultural information [ ], Reduces the need to travel for information [ ], Aids in obtaining agricultural information quickly [ ], Helps in networking with other farmers [ ], Increases farm productivity and income [ ]

Do you receive government agricultural extension services through?

Radio programs [ ], TV programs [ ], Mobile phone (Calls, SMS, Apps) [ ], None of the above [ ]

Do you receive agricultural support from any development organization via:

Radio programs [ ], TV programs [ ], Mobile phone (Calls, SMS, Apps) [ ], None of the above [ ]

Have you received any training on how to use radio, TV, or mobile phone for agricultural information?

government extension services [ ], NGOs or development organizations [ ] No, but I am interested [ ] No, and I am not interested [ ]

Item	Radio	Mobile Phone	Television
**Is the information from this type of media timely?**			
Timely			
Not Timely			
**Is the information from this type of media reliable for farming?**			
Reliable			
Not Reliable			
**Is the information from this type of media easy to use?**			
Easy			
Not Easy			
**How does the cost of using this information source compare to your usual source of information?**			
Same			
Lower			
Higher			
**Initial cost of buying the equipment for the media**			
**Cost of getting information from the media/month**			
**Types of services used to support agricultural processes**			
Radio/TV Program			
Radio Call-in			
Adverts			
Mobile Call-up			
Mobile SMS			
Mobile agri-based application			
Internet searching			
Mobile money services			

Note: These questions are based on your main devices you use for agricultural purposes.

Sustainable Agriculture

Kindly tick the SAPs you have adopted on account of participation in Savings group and those adopted without participation.

S/n	Category	On account of participation	Without Participation
**D1**	**Land management**		
D11	Limiting tilling and hoeing		
D12	No pasturing cattle in production areas		
**D2**	**Crop management**		
D21	Seedlings controlled diseases by chemicals before growing		
D22	Use of improved seed varieties		
D23	Irrigation		
D24	Rotation of crops		
D25	No-chemical weed control		
D26	Application of indigenous knowledge		
D27	Crop intercrop (legumes or annual crops)		
D28	Mulches		
**D3**	**Pesticide practices**		
D31	Biological pest and diseases control		
D32	Proper use of pesticides		
**D4**	**Fertilizer practices**		
D41	Application of inorganic fertilizers		
D42	Application of organic fertilizers (green and animal manure)		
D43	Conducting soil test before applying fertilizers		
**D5**	**Harvest management**		
D51	Cleaning production after harvesting		
D52	Using equipment for effective harvest		
D53	products harvested at maturity		
**D6**	**Post-harvest and marketing**		
D61	Products properly preserved and packaged		
D62	Products sold to enterprises through contractual agreement		
D63	Products registered with label showing ‘met quality standards’		
D64	Regular products quality checks by relevant authorities		
D65	Price setting using market price		
D66	Basic bookkeeping		

FOOD SECURITY

Kindly tick the foods you consumed in the last seven (7) days before the interview

cereals (tubers and white roots) [ ] vegetables[ ] fruits[ ] poultry and meat[ ] eggs[ ] fish[ ] legumes, pulses and nuts[ ] milk and milk products[ ] oils and fats[ ] honey and sugar[ ]spices, beverages, and condiments[ ]

What is your the weekly expenditures on mealie meal, rice, potato, vegetables, fish, meat, fruits, beverages and grocery items such as spices, onion, garlic, sugar and salt? **ZMW ____**

Household Food Insecurity Access Scale (HFIAS) Measurement Tool. Please only write the code in the spaces provided.

QUESTIONS	CODE	
	**ANSWER**<br>**0** = No<br>**1**=Yes	**IF YES, HOW OFTEN DID THIS HAPPEN?**<br>**1** = Rarely (once or twice in the past four weeks)<br>**2** = Sometimes (three to ten times in the past four weeks)<br>**3** = Often (more than ten times in the past four weeks)
In the past four weeks, did you worry that your household would not have enough food?		
In the past four weeks, were you or any household member not able to eat the kinds of foods you preferred because of a lack of resources?		
In the past four weeks, did you or any household member have to eat a limited variety of foods due to a lack of resources?		
In the past four weeks, did you or any household member have to eat some foods that you really did not want to eat because of a lack of resources to obtain other types of food?		
In the past four weeks, did you or any household member have to eat a smaller meal than you felt you needed because there was not enough food?		
In the past four weeks, did you or any other household member have to eat fewer meals in a day because there was not enough food?		
In the past four weeks, was there ever no food to eat of any kind in your household because of lack of resources to get food?		
In the past four weeks, did you or any household member go to sleep at night hungry because there was not enough food?		
In the past four weeks, did you or any household member go a whole day and night without eating anything because there was not enough food?		

Date of interview: ..../........./2022

THE END
THANK YOU SO MUCH FOR YOUR PARTICIPATION.
